# Inseparability between public health, planetary health and the nursing process: premise for sustainable development

**DOI:** 10.1590/1980-220X-REEUSP-2024-0026en

**Published:** 2024-06-28

**Authors:** Dirce Stein Backes, Roseléia Regina Halmenschlager, Talita Portela Cassola, Alacoque Lorenzini Erdmann, Kerstin Hämel, Regina Gema Santini Costenaro

**Affiliations:** 1 Universidade Franciscana, Programa em Saúde Materno-Infantil, Santa Maria, RS, Brazil.; 2 Universidade Federal de Santa Catarina, Programa de Pós-Graduação de Enfermagem, Florianópolis, SC, Brazil.; 3 Universität Bielefeld, School of Public Health, Bielefeld, Germany.

**Keywords:** Nursing Process, Community Health Nursing, Sustainable Development, Environmental Health, Nonlinear Dynamics, Proceso de Enfermería, Enfermería en Salud Comunitaria, Desarrollo Sostenible, Salud Ambiental, Dinámicas no Lineales, Processo de Enfermagem, Enfermagem em Saúde Comunitária, Desenvolvimento Sustentável, Saúde Ambiental, Dinâmica não Linear

## Abstract

The aim is to conduct theoretical reflection on the inseparability among public health, planetary health and the nursing process in light of complexity thinking, with the aim of contributing to healthy and sustainable development. Study with a theoretical-reflexive approach that accessed bibliographical sources from contemporary authors who defend the inseparability between public health and planetary health and, at the same time, provide theoretical-systemic support to the nursing process, under an inductive critical bias. The nursing process is conceived as a complex phenomenon, which comprises interdependent dynamics, dialogical approaches, critical-reflective perception and prospective leadership. Theoretical reflection on the nursing process and sustainable development raises an expanded, contextualized and interdependent look at the role of nursing professionals in different health contexts, in order not to compromise well-being and environmental health.

## RESUMO

Objetiva-se conduzir reflexão teórica sobre a indissociabilidade entre saúde pública, saúde planetária e processo de enfermagem à luz do pensamento da complexidade, no intuito de contribuir para o desenvolvimento saudável e sustentável. Estudo de abordagem teórico-reflexiva que acessou fontes bibliográficas de autores contemporâneos que defendem a indissociabilidade entre saúde pública e saúde planetária e, paralelamente, conferem sustentação teórico-sistêmica ao processo de enfermagem, sob um viés crítico indutivo. Concebe-se o processo de enfermagem como fenômeno complexo, que compreende uma dinâmica interdependente, abordagens dialógicas, percepção crítico-reflexiva e liderança prospectiva. A reflexão teórica acerca do processo de enfermagem e o desenvolvimento sustentável suscita um olhar ampliado, contextualizado e interdependente sobre a atuação do profissional de enfermagem nos diversos contextos da saúde, a fim de não comprometer o bem-estar e a saúde ambiental.

## RESUMEN

El objetivo es realizar una reflexión teórica sobre la inseparabilidad entre la salud pública, la salud planetaria y el proceso de enfermería a la luz del pensamiento de la complejidad, para contribuir con el desarrollo saludable y sostenible. Se trata de un estudio con enfoque teórico-reflexivo que accedió a fuentes bibliográficas de autores contemporáneos que defienden la inseparabilidad entre la salud pública y la salud planetaria y, al mismo tiempo, otorgan apoyo teórico-sistémico al proceso de enfermería, bajo un sesgo inductivo crítico. El proceso de enfermería se concibe como un fenómeno complejo, compuesto por dinámicas interdependientes, enfoques dialógicos, percepción crítico-reflexiva y liderazgo prospectivo. La reflexión teórica sobre el proceso de enfermería y el desarrollo sostenible suscita una mirada ampliada, contextualizada e interdependiente sobre la actuación profesional de enfermería en los diversos contextos de salud, con el fin de no comprometer el bienestar y la salud ambiental.

## INTRODUCTION

Understanding sustainable development leads to a path that considers current pressing needs and how these must be met so as not to compromise the well-being and health of future generations. Held in 2015, with the participation of 193 countries, the Sustainable Development Summit adopted 17 Sustainable Development Goals (SDGs) to be achieved by 2030. Also called Global Goals, they address the threats of climate change in a comprehensive and systemic way as people’s well-being and planetary health, based on economic, political, social and environmental dimensions^(1,2)^.

Increasing climate change and the degradation of ecosystems represent irreparable consequences for public health, which is inextricably linked to planetary health. The impact of these changes is associated with intense droughts, floods, heat waves, rising sea levels and others, the repercussions of which are highly harmful to humanity and the survival of biodiversity. Studies show, in this direction, that planetary health is already compromised and health threats already influence survival conditions, as well as human relationships, interactions and associations in the process of healthy living, especially in contexts of greater social vulnerability^(3-6)^.

These discussions, however, are not new to nursing, which for over 100 years has made an important contribution to the development of healthy and sustainable environments for people’s health and well-being. Although recognized as the philosophical founder of modern nursing, Florence Nightingale was also one of the most successful theorists in expanding the concept of health and inducing good practices related to environmental health, such as hygiene, cleaning, sanitation and others^(7,8)^.

Nursing, as a protagonist and reforming agent of health care, is driven to face new diseases and/or a growing number of cases of arboviruses resulting from climate change and ecologically irresponsible practices that degrade human, social, affective and emotional relationships^(9-12)^. It is essential, therefore, that nursing considers sustainable development in undergraduate and postgraduate curricula, also developing permanent education mechanisms focusing on the skills and competencies required for thinking and acting that are inseparable between public health and planetary health.

The nursing process, recognized as the standard of care practice, encompasses a circular and prospective movement that requires, on the part of nursing, critical-reflective perception, multidimensional and interprofessional theoretical alignment, in addition to an ethical, civic and visionary attitude. Based on theoretical support, the nursing process enables, through assessment, diagnosis, planning, implementation and evolution, an attentive, responsible and sustainable look at the practice of professional nursing practice^(13)^.

Considering this, the present study has its origins in the analysis of recent indicators that demonstrate the rapid and accelerated increase in climate change, the consequences of which could be devastating for the survival of biodiversity. Therefore, the research question should be: How can we conduct inseparable processes between public health, planetary health and the nursing process, in order to ensure healthy and sustainable development, from an integral and ecosystemic perspective? The objective, therefore, is to conduct theoretical reflection on the inseparability between public health, planetary health and the nursing process in the light of complexity thinking, with the aim of contributing to healthy and sustainable development.

In this field of discussion, the SDGs provide clear and prospective guidelines for the nursing process. Based on prospective theoretical support, the nursing process in the light of complexity thinking is not restricted to the interdependent stages - assessment, diagnosis, planning, implementation and evolution, but expands in the circular and inseparable understanding of the phenomena of care, health, well-being^(14)^. Based on this understanding, a specific stage of the nursing process will not be adopted in this study, but reflections will be conducted that require, from nursing, a systemic understanding of reality, in order to enable healthy and sustainable interventions in different scenarios.

## METHOD

This study has a theoretical-reflexive approach that accessed bibliographical sources from contemporary authors who defend the inseparability between public health and planetary health and, at the same time, provide prospective theoretical-systemic support to the nursing process, under an inductive critical bias. The inseparability between public health, planetary health, and the nursing process, in light of complexity thinking, provides a multidimensional perspective to sustainable development and, simultaneously, privileges cultural diversity, solidarity, ethics, equity, justice and human and social rights.

Complexity thinking, from Edgar Morin’s perspective, does not predict a linear path of conception and analysis of social and environmental phenomena^(15,16)^, but rather leads to a prospective itinerary, in which the researcher must lead his own path, from its context and lived experiences. Under this impulse, the proponents of this study focused, initially and individually, on the search and deepening of references that could broaden the theoretical perspective in relation to the object of investigation. After this first stage, meetings were held for socialization, discussion and refinement of reflective themes, which will be discussed below.

Conceived under this thought, the present study conceives the complexity of the nursing process and sustainable development in an expanded perspective, by weaving together the experiences of learning, teaching, investigating, congregating and reflecting on the impact of environmental degradation that leads to degradation of human, social, affective and emotional relationships. It is argued that inducing complexity thinking among nursing/health professionals is essential to an expanded, contextualized, sustainable and interdependent understanding of the stages of the nursing process^(17)^. To this end, the following reflective themes are explored without considering them conclusive: Thinking about complexity as an inducer of inseparable processes; and Nursing process as a generator of sustainable good practices.

### Thinking about Complexity as an Inducer of Inseparable Processes

It is assumed that complexity thinking is, par excellence, an inducer of circular and inseparable processes, be they public health, planetary health, the nursing process and others. Complexity thinking incorporates the notion of sustainability and defends the importance of the universal and the particular, the singular and the multidimensional, the individual and the collective, from an integral and ecosystemic perspective^(15)^.

Public health is configured as a complex unit, which is woven together, from the one and the multiple^(16)^. Interconnected with economic, political, cultural and environmental systems, health must be understood as a social good, not reducible to a marketing compound of production and dissemination of diseases and pharmaceutical resources. As a complex unit, health is interconnected with different social systems that aim to promote healthy living for individuals, families and communities, from an evolutionary and sustainable socio-ecosystemic perspective^(18)^.

The concept of planetary health emerged recently due to the intensification of the so-called greenhouse effect and consecutive global warming. It reflects the interdependencies between the health of the planet’s natural systems and the health of human civilization. Driven by modernity, which had unlimited progress at its heart, scientific and technological development promised the dreamed better future. This Eurocentric character of modernity and power imbalances between the global north and the global south, however, has been questioned and confronted in the light of references that reflect the ontological superiority of this devastating thinking and acting^(19)^.

The nursing process, in turn, transcends specific and linear technical perspectives. It reflects a professional way of being and acting committed to sustainable development. The nursing process therefore comprises an interactive dynamic, horizontal and dialogic approaches, critical-reflective perception and prospective leadership. Nursing professionals, especially nurses, need to be able to exercise circular and interdependent movements, in order to understand the unity in diversity, the whole in the part and vice versa^(20)^.

The principles of complexity thinking, including non-linearity, transdisciplinarity, open systems analysis and global-local phenomenology, provide a substantial theoretical basis for the inseparable understanding of the phenomena of public health, planetary health and the nursing process. These premises are in line with the multiple contexts of research, practice and education, whose limits are multidimensional, complex and, par excellence, induce good sustainability practices^(21)^.

Complexity thinking comprises a continuous process of (re)organization, resulting from the connection between different elements and dimensions that converge and determine health/care - a complex unit. In order to understand the world in its prospective and evolutionary dynamics, it is necessary to conceive a complex, open, organized mind, capable of contextualizing, making it more flexible, confronting certainties and analyzing facts and social events in a multidimensional, ethical and responsible way^(18-22)^. The human being with an open mind - a well-rounded head in Edgar Morin’s logic^(16)^ is capable of transcending rigid, one-dimensional and uncritical work processes, hegemonic in the simplified, reductive, involuted and devastating thinking of natural goods.

The dissemination of complex thinking among health professionals and citizens, in general, is urgent to achieve good sustainability practices in different areas and social contexts. Studies show that higher levels of complex thinking among people are associated with more favorable processes of communication, management, security and healthier and more sustainable human and social relationships. The favorable association between complex thinking and the promotion of preventive and protective health measures was also evidenced^(23,24)^.

Health, as a complex phenomenon, depends on the social structure and is determined by conditions that can be integrated into a tripod of sustainable development: economic development (economic growth, combating poverty, reducing social inequalities); social development (demography, economy and income, women’s empowerment, education, governance, health system structure); and environmental protection (geography, basic sanitation, safety, sustainable energy sources and others). From this perspective, the promotion of planetary health is the result of human activity that includes efforts to (re)construct transdisciplinary, inseparable and multidimensional educational processes^(25,26)^.

Associated with consumerist ideology, the economic model centered on productivity at any cost and discarding goods without criteria has reached its limits. Especially in the global south, it has reached its limits but with increasing impacts in the global north, degrading levels of survival and it has become unfeasible to follow the normative course without thinking about sustainable alternatives, such as recycling and adequate processing of waste, sustainable agriculture for food security, the preservation of natural habitats, the restriction of the use of pesticides, the protection of water resources and others, which directly impact the sustainability of human life and coexistence with nature. Systems capable of interacting and locating themselves in this complex thinking – evolutionary and sustainable – will be capable of fecundity, survival and sustainability of human, social, political and planetary relationships.

Instead of being recipients, the climate crisis calls us to thinking autonomy, to the conscious use of natural resources, to collegial, conscious and responsible leadership and to the promotion of the economy with a more human, fraternal, supportive and sustainable face. Economic and social transformation demands, in short, a new way of being and living in community, as well as courage to induce disruptive paths and rethink individual and collective choices towards healthy and sustainable development.

### Nursing Process as a Generator of Sustainable Good Practices

Evolutionary-sustainable thinking, from the perspective of complexity thinking, leads to coherence of life in the personal, professional and social spheres. This way of thinking translates into an emancipatory movement that moves from individual thinking to collective and collaborative action, in favor of human dignity and the survival of biodiversity. Organized into interrelated, interdependent, recurrent and cyclical stages, the nursing process^(13)^ transcends the vertical theoretical-interventionist perception and reveals itself as an inductive and (re)ordering alternative for a new way of thinking and proceeding in relation to care in health.

Considered one of the professions most involved in promoting health, nursing plays a central role in building a fairer, more prosperous and sustainable world for present and future generations. Global objectives are increasingly pursued by nursing by ensuring completeness and quality of access to health care, especially for the most vulnerable; by reducing disparities in access; by combating social and gender inequalities; by defending the construction of more equitable and peaceful societies; by contributing to the reduction of excessive health expenditures, by promoting the well-being and healthy living of individuals, families and communities^(27-29)^.

Good sustainable practices, in the field of nursing, include investments that range from the preservation of environmental resources and health-promoting habitats to simple changes in processes, behaviors and professional attitudes. Nursing professionals, through the nursing process, contribute to the well-being of users and easily adapt to adverse situations, in order to be decisive in referrals, optimize the work process and minimize travel and unnecessary interventions and others^(30)^.

With the aim of promoting shared management and comprehensive health care, especially in contexts of greater social vulnerability, nursing induces new ways of thinking about health and leads to more collegial movements that are synchronized with the common good. Studies demonstrate that ethical, conscious and integrated behaviors on the part of health professionals lead to higher levels of satisfaction and well-being of users, in addition to adding greater effectiveness and sustainability to the health care process^(31,32)^.

Faced with the growing climate crisis, professionals in general are challenged to develop strategies that contribute to reducing the emission of greenhouse gases and other pollutants that impact human and environmental health. In this sense, Chart 1 presents reflective questions that induce good sustainable practices in the nursing process. Developed based on scientific evidence^(33-35)^, these prospective questions aim to contribute to the responsible and sustainable use of resources in health services.

**Chart 1:** Reflective questions that induce sustainable good practices in the nursing process. Santa Maria, RS, Brazil, 2024.

N	Good sustainable practice	Reflective and evolutionary questions
1	Correct waste disposal	How do I segregate healthcare waste? How do I dispose of waste for possible reuse? How do my actions contribute to ensuring that discarded material does not have an impact on water, soil and air pollution? How do I contribute to optimizing resources and reducing expenses for users and the health service?
2	Reduction in paper consumption	What mechanisms do I use to separate the categories of paper that can be recycled? What is my position regarding digital platforms that aim to reduce the number of printed materials? What is my speech regarding the digitization of documents and data storage, with a view to reducing the consumption of paper and printed material in healthcare?
3	Rational water consumption	How do I avoid wasting water when washing hands? What is my attitude towards correct hand washing, in order to reduce cross-contamination? What is my attitude towards bathing with a view to reducing the amount of water being released into tributaries?
4	Conscientious preparation and administration of medications	What mechanisms do I use when preparing medications, in order to avoid errors related to dosage, waste and contamination? What precautions are eligible when administering medications, to avoid possible errors related to the route, patient and technique?
5	Use of biodegradable materials	What is my stance on purchasing biodegradable cleaning supplies? What is my practice regarding the use of reusable cups for coffee and water consumption?
6	Health promotion	What is my understanding of health and well-being, especially in relation to the most vulnerable groups? What strategies do I implement to promote health and reduce hospital admission rates for preventable reasons? What mechanisms do I encourage to promote health education and reduce hospital readmissions?
7	Ecological architectures in healthcare	What are my thoughts in relation to ecological architectures in healthcare, combined with reducing the consumption of energy, water, air conditioning and others? What initiatives do I promote in relation to the ecological environment and the evolutionary and sustainable nursing process?

A recently carried out study analyzed environmentally sustainable actions in the medication process, from receipt from the pharmacy to the disposal of waste by nursing professionals. The study identified a 74.8% reduction in chemical, infectious and sharps waste and a 48% reduction in waste sent to landfill, after educational nursing interventions^(33)^. These data reveal that the nursing process needs to intuit critical-reflective reasoning through systematic continuing education activities with the involvement of all actors.

Effective Continuing Education Programs are, therefore, capable of promoting greater engagement among nursing professionals and reducing the consumption of water, energy and toxic waste and, consequently, increasing the volume of recyclable materials. Continuing health education, based on interactive and associative theoretical-methodological references, such as complexity, are capable of intuiting evolutionary thinking, autonomous and committed to good sustainable practices in health^(36)^.

The containment measures imposed by the Covid-19 pandemic also demonstrate that nursing can go beyond normative and institutionalized practices, more specifically with regard to virtual consultations. A study shows that between March 2020 and April 2021, greenhouse gas emissions were reduced by more than 15 thousand metric tons. Furthermore, 5.3 million printed pages were eliminated due to digital programs and virtual consultations promoted in health services^(37)^. These and other measures demonstrate that it is possible to overcome traditional practices and invest in new approaches that guarantee greater sustainability in health services.

The nursing process, under this impulse, must/can be understood as a complex phenomenon, based on multiple dynamic relationships, interactions and systemic associations, with a view to promoting human, social and environmental well-being^(19)^. Due to its human, interactive and proactive nature, nursing is capable of intuiting good sustainable practices in different health contexts, as long as there is motivation and engagement on the part of professionals.

Morin argues that it is necessary to teach human understanding based on transdisciplinary mechanisms correlated with the world in movement. It is important to consider, in this sense, that the living systems that make up society are in permanent evolutionary dynamics and producing changes that require contextualized analysis on the part of citizens. Under this approach, Morin stands for an integral, transdisciplinary and systemic thinking, capable of questioning the mutilating thinking that only captures a part to satisfy local and transitory interests^(15)^.

By questioning the predictable, the absolute and the linearity of human actions, the thought of complexity provides the opportunity to rethink planetary reality through the ability to bring together, contextualize, globalize, but also to recognize the singular and multidimensional in relation to sustainable development^(15,16)^. Achieving a systemic perspective in relation to sustainable development means realizing and promoting interactivity, complementarity and sustainability with and among all things and creatures.

Conceiving the nursing process as an inducer of good sustainable practices necessarily implies expanding the conception of human beings, citizenship, care, health, health management/leadership, and healthy living. If the nursing professional has the skills and potential to promote the nursing process as a common-social good, they also have the skills to evolve, (re)construct, aggregate, innovate and prospect sustainable strategies and policies committed to life and health^(38)^.

The contributions of this study to the advancement of nursing science are related to the proposition of complex thinking that induces expansion, complementarity and inseparability between public health, planetary health and the nursing process. Another contribution is associated with the promotion of evolutionary and sustainable thinking among nursing professionals, capable of expanding and inducing sustainable good practices in different health environments and contexts.

The proposition of only one theoretical framework – complexity thinking to discuss the nursing process and sustainable development, constitutes a limitation for the advancement of knowledge in this specific area. Another limitation may be associated with the fact that this study did not consider a specific stage of the nursing process, but rather the dynamicity of each part as a whole. However, there are several views for reflection that can be considered by other thinkers/researchers to advance in the deliberation of new perspectives that will induce good sustainability practices in nursing/health.

## CONCLUSION

Theoretical reflection on the nursing process and sustainable development raises an expanded, contextualized and interdependent look at the role of nursing professionals in different health contexts, in order not to compromise well-being and environmental health. In an incipient form, theoretical reflection suggests nursing interventions related to the sustainable use of natural resources and greatly highlights the importance of continuing to deepen the close interconnection among social, environmental and health factors.

Understanding the actions that induce good sustainable practices and the promotion of care associated with planetary health are important challenges to be overcome for the advancement and sustainability of nursing science. Deliberated in a systematic and prospective manner throughout the socio-environmental context in which nursing care occurs, the nursing processes are able to promote evolutionary strategies and policies aligned with the achievement of the Global Objectives.
